# Commentary: No unique effect of intergroup competition on cooperation: non-competitive thresholds are as effective as competition between groups for increasing human cooperative behavior

**DOI:** 10.3389/fpsyg.2017.02322

**Published:** 2018-01-10

**Authors:** Bonaventura Majolo, Teresa Romero

**Affiliations:** ^1^School of Psychology, University of Lincoln, Lincoln, United Kingdom; ^2^School of Life Sciences, University of Lincoln, Lincoln, United Kingdom

**Keywords:** behavioral economics game, between-group competition, cooperation, conflict, threshold, war

Between-group competition has been proposed to affect the occurrence of cooperation. Within-group cooperation should increase under between-group competition, as cooperation increases the chances of winning the competition, other things being equal (e.g., group size; Bowles, 2009; Van Vugt and Park, 2009). In humans, studies testing this hypothesis have often used public goods games where participants are assigned to different groups and have to decide whether/how much of their allocated resource they want to contribute to their group (e.g., Puurtinen and Mappes, 2009). Each participant's winnings/losses depend on the amount of resources that they kept to themselves and on the total group contribution. In the between-group competition condition of the game, the group displaying the greatest within-group cooperation (i.e., total group contribution), in comparison to the competing groups, wins the competition and gets an extra reward, shared equally among the group members. Supporting the hypothesis, within-group cooperation is greater in the between-group competition than in the no-competition condition (Puurtinen and Mappes, 2009; Burton-Chellew and West, 2012; Puurtinen et al., 2015; Majolo and Maréchal, 2017).

Jordan et al. (2017) point out that the design of the between-group competition experiment creates a threshold effect. According to this argument, the total amount of cooperation done by each group (i.e., their total group contribution) sets a threshold that needs to be crossed by the competing groups in order to win the extra reward. If so, such experimental approach cannot disentangle the relative importance of between-group competition and threshold effects on cooperation. The mere presence of a threshold might increase cooperation because groups are trying to reach the threshold set by their opposing groups, not because of the actual between-group competition.

Jordan et al. (2017) shows that the presence of a reward (monetary or else) increases within-group cooperation. However, we argue that thresholds are an inherent feature of any competitive interactions and no competition can exist in nature without a threshold. Therefore, the artificial separation of competition and threshold in laboratory conditions has a limited impact in our understanding of the evolution of cooperation. The outcome of any competitive interaction depends on what each competing agent does and on the relative performance and competitive powers of the competing agents. That is, whether the actions of one agent outperform those of the other competing agents. The relative performance and competitive power of the opposing agent(s) determines the threshold that each agent needs to reach to gain access to the contested resource. Thus, the question to ask is: are there competitive interactions in nature that are threshold-free? The answer to this question is, in our view, no.

Competition can involve either depletion of the same resource at different times, with no direct interactions between agents (scramble competition), or direct aggressive confrontation between competing agents (contest; e.g., Isbell, 1991; Henson and Cushing, 1996). In scramble competition, what matters is whether an agent can find and deplete a resource before the other agents, whilst potentially avoiding aggressive interactions at the same time. The outcome of contest competition does not depend on the absolute fighting ability of each competing agent but on their relative ability in comparison to that of their opponent. Providing that the motivation to fight is fixed and the same for all the competing agents, the threshold that each competing agent needs to reach to win the competition is set by the fighting ability of their opponents. This threshold can vary across opponents and/or during a single competitive interaction, as in sequential assessment games (Enquist and Leimar, 1983). Thus, the relative fighting ability of the agents competing for resources determines the selection pressure exerted on the agents in an arms race scenario (Dawkins and Krebs, 1979). Selection will favor agents who have slightly better competitive ability than their opponents instead of those who out-compete other agents by a great degree, because the latter will incur the unnecessary cost of growing/sustaining traits related to competition. For example, mating opportunities for a red deer male (*Cervus elaphusus*) depend of the size and complexity of their antlers in comparison to those of the opponents, not on the absolute size and complexity of their antlers (Malo et al., 2005). Thus, contest competition, including contests between groups, is not threshold-free. Furthermore, opponents aggressively compete with one another over rewards that would not be available if agents did not engage in the contest (e.g., Markham et al., 2012; Crofoot, 2013; Radford and Fawcett, 2014). Therefore, behavioral economics experiments on between-group competition must incorporate a reward that each group is competing for, to make these experiments ecologically valid.

We acknowledge that there are several cases in nature where thresholds exist outside competition and it is beneficial for an animal to reach such thresholds. For example, sleeping beyond a given height on a tree may give an animal security against large terrestrial predators. However, the key point is that, since competition and threshold are tightly linked together and cannot really be disentangled in the natural world, these two elements should have jointly exerted evolutionary pressure on social evolution. Thus, humans and other animals should not have evolved more/less “sensitivity” to threshold than to competition because these two are aspects of a single driving force, competition, with its associated costs and benefits for individual fitness.

In conclusion, selective pressure on competitive skills does not exists in a “vacuum” but depends on the relative competitive abilities of the competing agents which, in turn, set the threshold for each agent to out-compete the others. It is unlikely that different selective pressure has acted on sensitivity to thresholds and competition independently from one another. The role that between-group competition might have played on social evolution is still largely unclear, but resource maximization and response to competition are likely to be two intertwined biological phenomena.

## Author contributions

All authors listed have made a substantial, direct and intellectual contribution to the work, and approved it for publication.

### Conflict of interest statement

The authors declare that the research was conducted in the absence of any commercial or financial relationships that could be construed as a potential conflict of interest.
